# Robotic-assisted Roux-en-Y gastric bypass with the novel platform Hugo^TM^ RAS: preliminary experience in 15 patients

**DOI:** 10.1007/s13304-023-01657-7

**Published:** 2023-10-20

**Authors:** Marco Raffaelli, Francesco Greco, Francesco Pennestrì, Pierpaolo Gallucci, Luigi Ciccoritti, Giulia Salvi, Priscilla Francesca Procopio, Nikolaos Voloudakis

**Affiliations:** 1 U.O.C. Chirurgia Endocrina e Metabolica, Centro Dipartimentale di Chirurgia Endocrina e dell’Obesità, Fondazione Policlinico Universitario Agostino Gemelli IRCCS, Rome, Italy; 2 Centro di Ricerca in Chirurgia delle Ghiandole Endocrine e dell’Obesità, Università Cattolica del Sacro Cuore, Rome, Italy

**Keywords:** Hugo™ RAS, Roux-en-Y gastric bypass, Robotic surgery, Minimal invasive surgery, RYGB

## Abstract

Robotic assisted surgery is a rapidly developing field in bariatric surgery. Its wide diffusion has led to the development and standardisation of robotic assisted approaches for various bariatric operations. However, further application has been limited thus far due to issues of cost-effectiveness and accessibility. The introduction of new robotic platforms may help overcome those obstacles. In this study, we present the first fifteen cases of Roux-en-Y Gastric Bypass (RYGB) operations performed with the new Hugo™ RAS system (Medtronic, Minneapolis, MN, USA). From January to March 2023, consecutive patients scheduled for minimal invasive Roux-en-Y-Bypass were randomly selected and underwent the procedure robotic-assisted with the new platform. No exclusion criteria were applied. Seven female and eight male patients with a median BMI of 42 (range: 36–50) and obesity-related comorbidities in eight cases underwent RYGB. The median docking time was 7 min (range: 6–8.5) and the median console time was 100 min (range: 70–150). Procedures were performed without intraoperative complications and no conversion to laparoscopy or open surgery was noted. Operative times were indicative of a steep learning curve. No early post-operative complications were observed. Based on our initial experience, RYGB with the Hugo™ RAS system is promising and may be integrated in established robotic programmes without requiring a long adaptation period.

**Supplementary Information:**

The online version contains supplementary material available at 10.1007/s13304-023-01657-7.

## Introduction

Robotic assisted surgery has been steadily gaining ground over the last two decades in bariatric operations [1], whilst Roux-en-Y Gastric Bypass (RYGB) has been the most performed bariatric operation in Europe and is amongst the most popular in the world, particularly for coexisting gastroesophageal reflux disease [2]. Bariatric patients, and especially the super obese subgroup, present with technical challenges in minimal invasive surgery, due to the thick abdominal wall, enlarged liver, and increased visceral fat. Consequently, the working space and exposure is limited, while torque forces are amplified [3, 4]. In addition, a significant proportion of surgeons prefer the hand-sewn anastomosis, further leading them towards a more ergonomic robotic solution [1, 5, 6].

The further diffusion of robotic approaches is mainly hindered by platform accessibility and increased costs. Additionally, systematic reviews thus far in bariatric surgery in general [7, 8], and Roux-en-Y specifically [9, 10], have failed to prove systematic benefits of Robotic Assisted Surgery (RAS), thus rendering it less cost-effective.

One of the main solutions for the future of RAS in bariatrics lies in improving access and establishing a healthy-competitive market. Currently, there are several platforms announced for release, or under development, but only a limited number of them have been FDA approved or received a CE (Conformité Européenne) mark. In our Centre, which has an established robotic programme among various specialities and two DaVinci® Xi platforms (Intuitive Surgical ™), we have gained access to the novel platform Hugo™ RAS (Medtronic, Minneapolis, MN, USA) for nearly a year now. After our initial experience with adrenal operations, where the platform’s features were also described [11], we attempted to assess the performance in more challenging, multi-quadrant operations that also require hand-sewing for constructing an anastomosis. This was only possible after the platform received CE approval also for general surgery (October 2022). The safety and feasibility of the Hugo™ RAS platform has already been tested in urological [12, 13], gynaecological [14, 15], bariatric [16] and adrenal procedures [11], while the first report of its application in colorectal surgery has also been very recently reported [17]. However, there are no reports thus far, with enough clinical evidence, exploring if the transition to novel robotic platforms in robotic centres is an uneventful process, without requiring a long adaptation period.

The aim of the current study was to report our early experience with the Hugo™ RAS platform in bariatric procedures (RYGB) and to assess the outcomes of the first 15 cases.

## Methods

Between late January and March 2023, fifteen consecutive informed patients underwent robotic-assisted RYGB with the Hugo™ RAS system in our Institution, a tertiary referral centre for bariatric surgery. No specific exclusion criteria were applied for patient selection, apart from being scheduled for minimal invasive RYGB. Operations were performed by a surgeon experienced in both laparoscopic and robotic bariatric operations (M.R.). Participating surgeons and nurses had previously completed the technical training on Hugo™ RAS System delivered by Medtronic at the ORSI Academy, Aalst, Belgium and were familiar with the platform from performing other types of operations with the same system [11]. Informed consent of all participating patients was acquired.

## Surgical technique

### Patient position and trocar placement

Under general anaesthesia, the patients were placed in a supine position with the legs split (“French” position). Pressure points and bony prominences were padded for protection. Patients’ body was secured with a gel pad and strapped across the thighs to avoid any shifting in the reverse Trendelenburg position (approx. 15%).

**Fig. 1:**
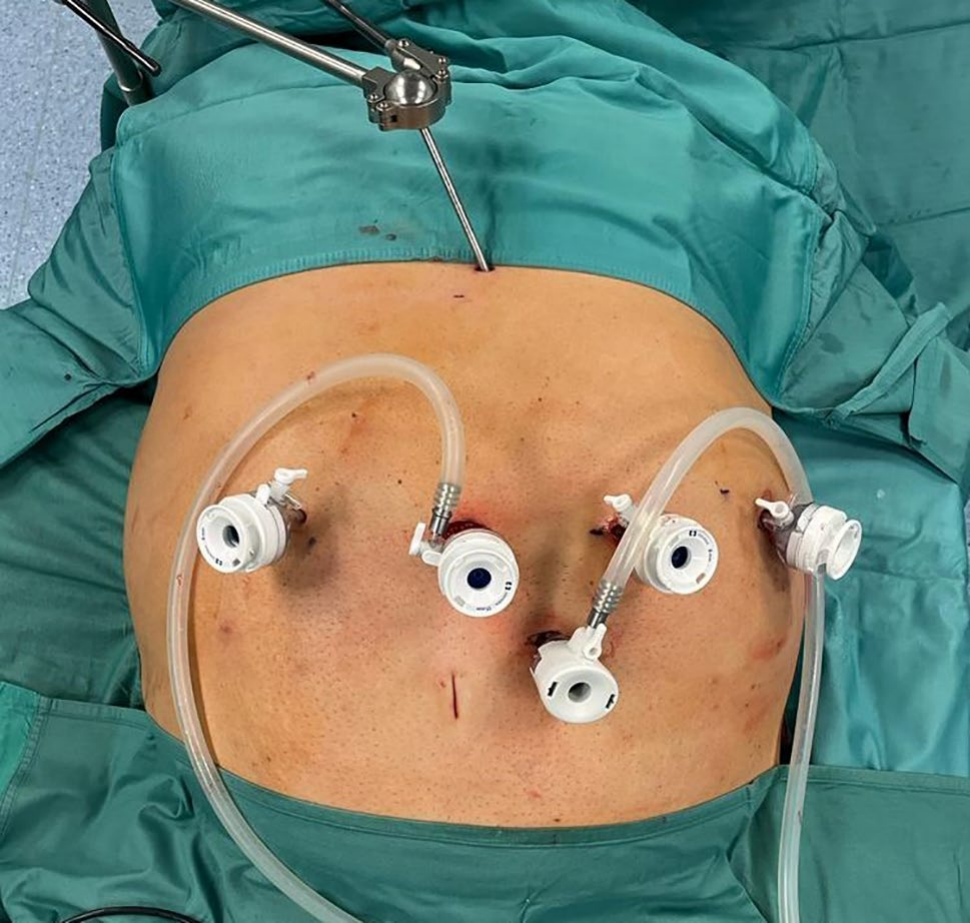
Trocar placement sites for robotic-assisted RYGB with the Hugo^TM^ RAS

Caution is advised when placing the robotic ports. A minimum distance of 8 cm between them is required to avoid collisions during the operation. The first port, an 11-mm camera port, was placed supraumbilically, slightly on the left at approximately 15 cm below the xiphoid process. Following pneumoperitoneum establishment, three 8-mm robotic trocars were then inserted, one on each flank and one in the left subcostal space, along a parallel line **(**Fig. 1**)**. A 12-mm accessory trocar, to be used by the assistant, was placed inferiorly, in the middle of the distance between the camera trocar and the surgeon’s right-hand trocar. Ultimately, a Nathanson’s liver retractor was inserted in the subxiphoid area.

### Docking

**Fig. 2:**
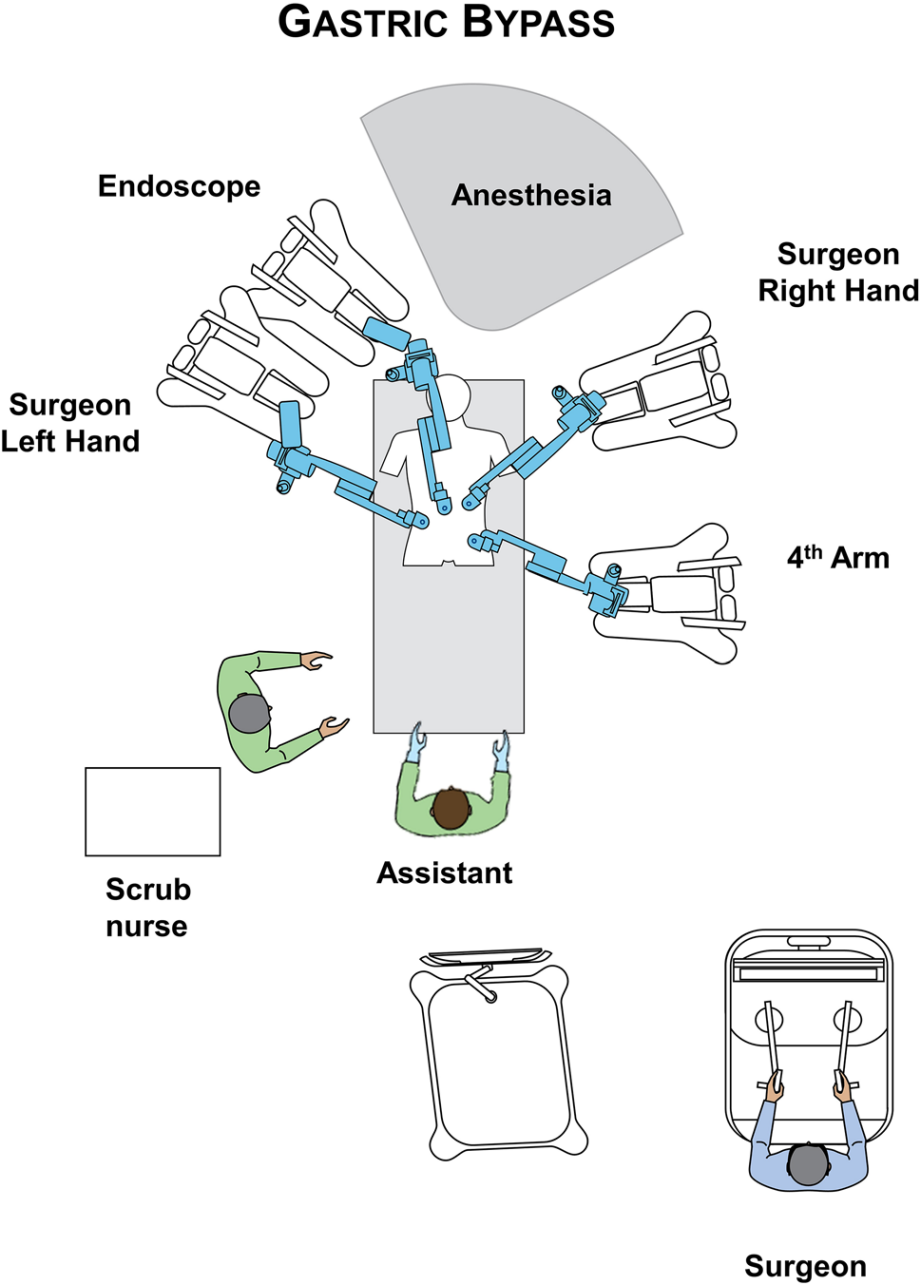
Operating room and docking settings for the RYGB procedure with the Hugo^TM^ RAS

The Hugo™ system consists of 4 independent arm carts. Each arm requires its own settings, that can be adjusted depending on the patient’s body type. Two main settings are required to configure each arm. One is the *tilt angle*, which is the vertical angle of the arm in respect to the flat operative bed (0°) and can be adjusted by lifting upwards or downwards the arm’s nose. The other is the *docking angle*, which is the clockwise horizontal angle between the head of the patient (0°) and the arm’s direction [11]. Configurations were defined by our team along with the company’s personnel prior to the operations on a surgical manikin. Adjustments in the settings of the third and fourth robotic arms were made to match the second patient’s body type, create more room for the anaesthesia personnel and avoid collisions. Suggested configurations are reported in in Fig. 2. The docking and tilt angle configurations that matched most body types for robotic assisted RYGB procedures were the following: endoscope: 355° and − 45°, surgeons left hand: 290° and − 30°, right hand: 40° and − 45°, and 4th arm 115° and 30°. Those configurations can be slightly adjusted depending on patient’s body features, and surgeon’s individual preferences.

It should be noted that, in contrast to the Da Vinci Xi™ system, the platform does not have a “memory” of the docking for each procedure and must be manually configured separately each time.

In all operations, a bipolar fenestrated grasper was used for the left surgeon’s hand, a monopolar curved sears (with protective tip cover) for the right, switched with a large needle-driver during the anastomosis construction. A secure Cadiere forceps or a double fenestrated grasper was used for the fourth robotic trocar.

### Operation

We applied the antecolic double-loop technique for the RYGB [18]. A short video of the procedure has been provided for the readers’ convenience. The first step was the creation of the gastric pouch. Following adequate exposure of the gastroesophageal junction, by retracting the left liver lobe with the Nathanson retractor, the lesser sac was entered along the lesser curvature approximately 6 cm from the oesophago-gastric junction and the stomach. The bed-assistant introduced the powered laparoscopic linear stapler (Signia™, Medtronic) through the accessory trocar. The gastric pouch was created using three purple cartridges (one horizontal, two vertical): a 36 F orogastric bougie was used for calibrations. A small bowel loop approximately 75 cm distally from the Treitz ligament was selected and brought upward in an antecolic fashion without tension. A robotic, hand-sewn, end-to-side gastrojejunal (GJ) anastomosis was performed with two layers running suture (Stratafix™ and PDS®, Johnson & Johnson, Medtech, USA) [19]. A second loop of small bowel, 150 cm from the GJ anastomosis alimentary side, was identified and brought up to perform an entero-enteric side-to-side stapled anastomosis (single bronze cartridge) between the second loop and afferent limb of the GJ anastomosis. The insertion holes were then closed with a running absorbable suture (Stratafix™). The integrity of the two anastomosis was verified with a blue methylene and pneumatic test through a nasogastric tube positioned in the efferent limb (only the GJ anastomosis tests are visible in the video). A gauze placed upon and behind the GJ anastomosis was then checked by the surgical staff for presence of methylene blue. The last step was the creation of the Roux-en-Y, by dividing the jejunum between the two anastomoses via a linear stapler (single bronze cartridge, Signia ™). A drain was placed posteriorly to the GJ anastomosis.

### Post-operative protocol

A standard postoperative protocol, personalised for bariatric patients was used. All patients remained nil per OS until an upper gastrointestinal (UGI) hydrosoluble contrast (Gastrografn®, Bracco SpA, Milan, Italy) study was performed on the first post-operative day [20]. Liquid diet commenced after the UGI contrast study, if no leak was observed, and clinical course was uneventful. Routine complete blood examination and blood count were obtained on 1st post-operative day in all patients. The severity of postoperative complications was rated according to the Clavien–Dindo classification [21]. Patients were discharged 24 h after the surgical procedure if the following conditions were met: no clinical complications or postoperative biochemical and imaging alterations occurred; oral alimentation was tolerated; autonomous in life activities; the discharge was accepted by the patient. The complete post-operative and follow-up protocol has been previously described in detail and is beyond the scope of this study [22, 23].

## Results

**Fig. 3:**
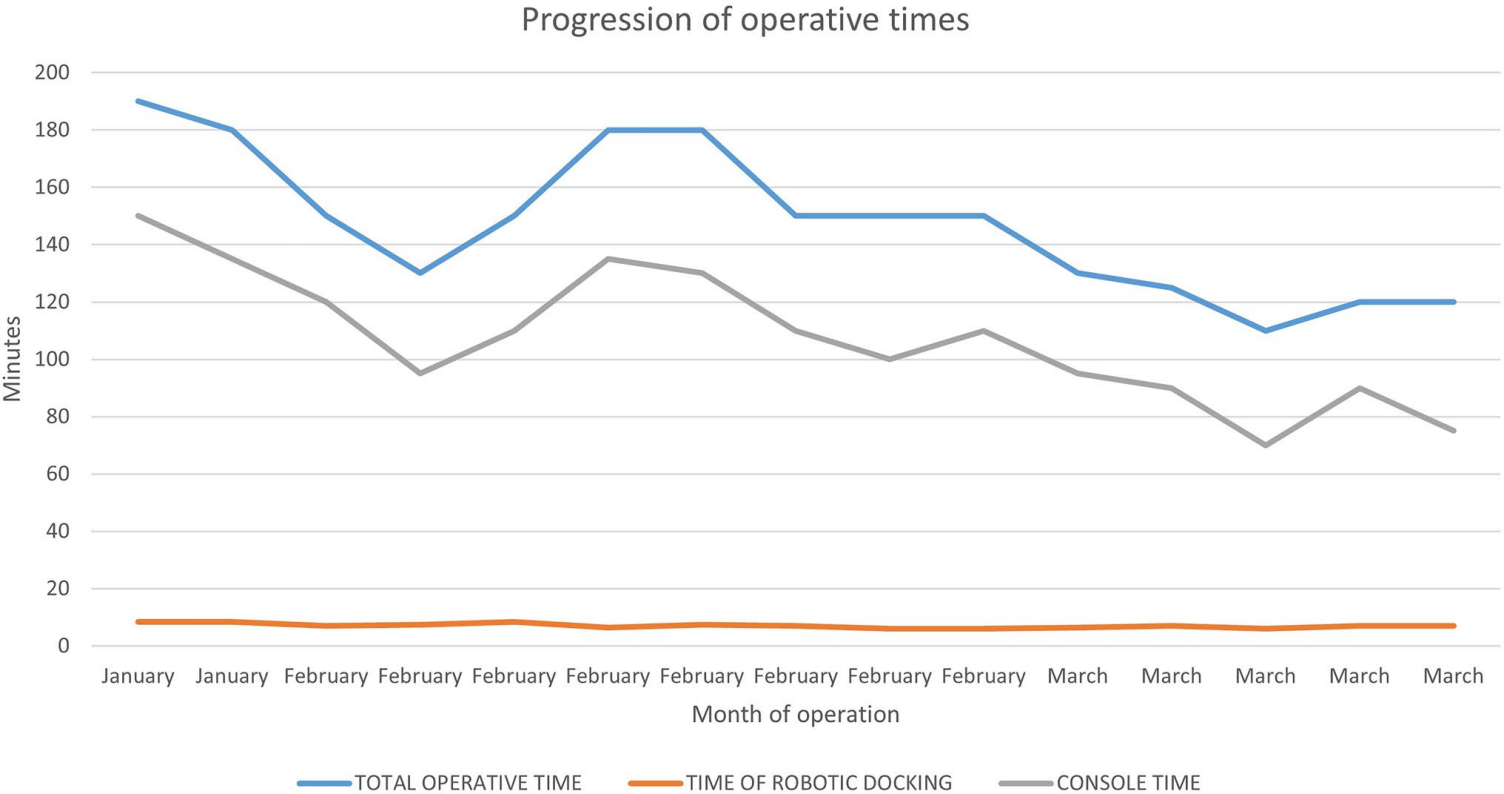
Progression of operative times between patients operated for RYGB with the Hugo^TM^ RAS platform

Seven female and eight male patients underwent robotic-assisted RYGB with the Hugo™ RAS platform. Patients’ characteristics, operative details, and post-operative course are shown in Table 1. There were no intraoperative complications or system failures. All operations were completed without additional port placement or conversion to either laparoscopic or open surgery. The median console time was 110 min (range: 70–150), median docking time 7 min (Range: 6–8.5), while the median total operative time 150 min (Range: 110–190). As it is shown in Fig. 3, there was a steady reduction of all operative time parameters, potentially signifying that the surgical team’s adaptation to the new platform requires a very limited number of cases, if previously familiar with robotic RYGB and the new platform. The length of hospital stay ranged between one and three days in all cases (Table 1). Eight patients presented with obesity-related comorbidities, including 3 with OSAS, 4 with Hypertension, and 3 with diabetes.

**Table 1:** Patients’ characteristics, operative data, and post-operative course

	*n* = 15
Age (years) Median, range	48 (31–65)
Sex (Male/Female)	7/8
Weight (kg) Median, range	131 (97–183)
Height (cm) Median, range	168 (156–196)
Body mass index (Kg/m^2^) Median, range	42 (36–50)
Comorbidities (cases)	8
Previous abdominal surgery (open/laparoscopic)	4/2
Docking time (min) Median, range	7 (6–8.5)
Console time (min) Median, range	100 (70–150)
Total operative time (min) Median, range	150 (110–190)
Arm collision (cases)	2
Intra-operative complications	0
Post-operative complications	0
Length of hospital stay (days) Median, range	2 (1–3)

In the first operation, there were a few instances of clashing between the robotic arms extra-abdominally, namely between arm 3 and 4. This did not lead to any noteworthy time delay or adverse events, since there is a built-in alarm system that momentarily stops the instruments until the operator unblocks them manually. To avoid such occasions, the surgical team must first ensure that the distance of the robotic trocars is at least eight centimetres. Furthermore, small adjustments in carts placement, docking and tilt angles of the arms were made in order to provide more ample space for each arm extra-corporeally. Clashes between the robotic arms were also noted in the 8^th^ patient, potentially due to an extreme body type (156 cm height). Lastly, abrupt manoeuvres should be avoided. By applying those modifications, this issue was resolved.

## Discussion

In this study, we report on the outcomes of the first 15 cases of Roux-en-Y gastric bypass with the Hugo™ RAS, which were indicative of a particularly short learning curve when applied in bariatric centres with established robotic programs.

Since the first robotic-assisted surgery (RAS), a cholecystectomy, was performed in 1997 [24], RAS has slowly but steadily gained ground among most surgical fields. The protagonist, and until recently single player, of robotic solutions, Intuitive Surgical™, has led, and defined the market so far by providing constant improvements and several updated platforms, [25]. By the end of 2021, 6730 da Vinci Surgical Systems had been installed worldwide, including 4139 in the U.S., 1119 in Europe, 1.050 in Asia and 342 in the rest of the world. Approximately 1,594,000 surgical procedures were estimated to have been performed in 2021 alone, while since 2018 general surgery procedures became prevalent [26].

The perceived advantages of RAS include improved ergonomics, stereoscopic vision, hand tremor filtration, and instruments with more degrees of freedom [6, 25]. The above can potentially result in improved surgical dexterity and maximise surgical efficiency compared with conventional laparoscopic surgery. However, the main limitations of further RAS diffusion have been repeatedly associated with increased costs, longer operative times, and lack of platform accessibility. Published individual studies and meta-analyses have failed to systematically yield superior results of robotic-assisted RYGB in comparison to the laparoscopic conventional approach, thus associating robotic-assisted RYGB with reduced cost-effectiveness [9, 10, 27]. However, included studies were not randomised, varied in surgical techniques applied, and surgeons’ expertise in robotic procedures. Indeed, among comparative studies included in the pertinent systematic reviews and meta-analyses various biases may apply. One of them being that authors rarely report if included cases were performed after completing the learning curve for each procedure/approach which may have negatively affected the results mainly for the robotic arm [28].

In bariatric operations, the use of a robotic platform greatly facilitates the execution of a hand-sewn end-to-side gastrojejunostomy; even if the comparison between the mechanical and the manual technique has not shown substantial differences systematically, the hand-sewn anastomosis could have some advantages in terms of reduced risk of bleeding [29–31] and greater control of the gastric pouch emptying, allowing to better modulate the anastomosis calibre [32]. It has also been demonstrated that the hand-sewn anastomosis, even in laparoscopy, allows a more tailored approach, as opposed to a stapled one, potentially leading to less complication rates [33]. In addition, limiting the use of staplers, either robotic or laparoscopic ones, certainly reduces overall procedural costs [34].

However, the main costs are associated with the initial capital invested in platform purchase, the limited life span of instruments used, and annual maintenance. It has been thus estimated that to address and rationalise the increased costs of robotics with the Da Vinci platform, at least 250 procedures/year are necessary to be performed in a medical centre [27, 35]. It has been proposed that the financial burden of robotics may be alleviated by reducing the complications rates, readmissions, and operative times in the future, especially in revisional and challenging operations. Nonetheless, to-date, such projections have not been verified by published evidence [7]. A more promising solution would be mitigating costs by establishing a healthy competitive environment between companies involved in robotic surgery solutions. The introduction of novel platforms in the market is bound to create such an environment, potentially propelling a significant reduction in costs and further diffusing robotic technologies [25, 36].

Heading into the future, the competition among RAS platforms is expected to be fierce, since multiple companies have already released, or announced, their own robotic systems. Those include Asensus Surgical, Inc.; avateramedical GmbH; CMR Surgical Ltd.; Johnson & Johnson; Medicaroid, Inc.; MedroboticsCorporation; Medtronic plc; meerecompany Inc.; MicroPort Scientific Corporation; Olympus Corporation; Samsung Group; Titan Medical Inc, and others [26, 36]. The question, however, remains if the introduction of new platforms in already established robotic programs will be a smooth process, or if the differences between the robotic systems will lead surgeons to undergo a new lengthy “adjustment” period.

In our centre, although having an established bariatric robotic program since 2013, no more than 2% (≈ 100 cases) of all bariatric operations were able to be scheduled with such an approach. Due to limited platform availability, and economic concerns, RAS was mainly applied in more challenging cases such as patients with super-obesity, revisional surgery, RYGB and single anastomosis duodeno-ileal bypass with sleeve gastrectomy (SADI-S) [22, 27]. However, it should be noted that in this series, no patients with super-obesity or revisional surgery cases were included. This did not occur due to study design, as no such exclusion criteria were set, but randomly since the patients scheduled were consecutive. Nonetheless, this allowed the surgical team to assess the new platform’s performance and refine the specifications under most circumstances, but did not provide additional evidence on its potential benefits in more challenging cases.

Given the favourable results and potentially short adaptation period with the new Hugo™ RAS platform presented here, the prospect of concomitantly utilising both platforms in bariatric surgery seems very promising. Indeed, intraoperative, and post-operative complications in this series were zero, while operative times were well within both ours and internationally reported ranges with the DaVinci® platform [10, 27].

There are very few reports published commenting on the transferability of skills between robotic platforms. In a very recent work by Larkins et al. [37], ten participants sequentially completed four Mimic®(Surgical Science) simulation exercises on two different robotic operating platforms (DaVinci®, Intuitive Surgical and Hugo™ RAS, Medtronic). One group was allocated first on the one console and then performed the same exercises on the other one. The sequence was contrariwise for the second group. Both quality and efficiency metrics, as well as risk and safety metrics, were equivalent across groups, indicating that training in the two platforms can be performed concomitantly. From our early experience presented here, it is our view that experienced surgeons with the DaVinci® platform may easily adapt to Hugo™ RAS, perhaps signifying that robotic skills and experience gained may act in a cumulative fashion.

The beneficial impact of wider diffusion through multiple platforms and concomitant use, can be applied in the future also in robotic training programs. In a recent “snapshot” study [38] among all specialty surgical trainees, 73.8% (*n* = 180) of participants would value greater access to robotic surgery training, 73.4% (*n* = 179) believed that robotic surgery was important in their speciality future, 77.2% (*n* = 156) stated that it should be incorporated into formal surgical training, while a portion of the participants that had robotic programs in their hospitals, perceived a negative impact on their training due to consultant robotic learning curves. The introduction of multiple RAS platforms might address both the accessibility issue and shorten the relevant learning curves.

Although the number of cases reported here are limited, and it would be premature to perform a learning curve analysis, it is our belief that the future of RAS may lie in multi-platform robotic centres, which would offer new opportunities not only in broader diffusion of robotic assisted surgery, but also in robotic training. Nonetheless, larger series of procedures performed with the new platforms, as well as appropriately designed skills transferability reports, are necessary to validate our initial results.

### Supplementary Information

Below is the link to the electronic supplementary material.

Supplementary file1 (MP4 445779 KB)

## Data Availability

The data presented in this study are available on request from the corresponding author. The data is not publicly available due to privacy and ethical restrictions.

## References

[1] Bauerle WB, Mody P, Estep A (2022). Current trends in the utilization of a robotic approach in the field of bariatric surgery. Obes Surg.

[2] Ozsoy Z, Demir E (2018). Which bariatric procedure is the most popular in the world? a bibliometric comparison. Obes Surg.

[3] Gagner M, Gumbs AA, Milone L (2008). Laparoscopic sleeve gastrectomy for the super-super-obese (body mass index >60 kg/m(2)). Surg Today.

[4] Parikh MS, Shen R, Weiner M (2005). Laparoscopic bariatric surgery in super-obese patients (BMI > 50) is safe and effective: a review of 332 patients. Obes Surg.

[5] Bindal V, Sethi D, Pandey D (2021). Robotic primary bariatric surgery. Dig Med Res.

[6] Dalager T, Jensen PT, Eriksen JR (2020). Surgeons’ posture and muscle strain during laparoscopic and robotic surgery. Br J Surg.

[7] Bertoni MV, Marengo M, Garofalo F (2021). Robotic-assisted versus laparoscopic revisional bariatric surgery: a systematic review and meta-analysis on perioperative outcomes. Obes Surg.

[8] Zhang Z, Miao L, Ren Z, Li Y (2021). Robotic bariatric surgery for the obesity: a systematic review and meta-analysis. Surg Endosc.

[9] Economopoulos KP, Theocharidis V, McKenzie TJ (2015). Robotic vs. laparoscopic Roux-En-Y gastric bypass: a systematic review and meta-analysis. Obes Surg.

[10] Aiolfi A, Tornese S, Bonitta G (2019). Roux-en-Y gastric bypass: systematic review and Bayesian network meta-analysis comparing open, laparoscopic, and robotic approach. Surg Obes Relat Dis.

[11] Raffaelli M, Gallucci P, Voloudakis N (2023). The new robotic platform Hugo^TM^ RAS for lateral transabdominal adrenalectomy: a first world report of a series of five cases. Updates Surg.

[12] Totaro A, Campetella M, Bientinesi R (2022). The new surgical robotic platform HUGO TM RAS: system description and docking settings for robot-assisted radical prostatectomy. Urologia.

[13] Ragavan N, Bharathkumar S, Chirravur P (2022). Evaluation of Hugo RAS system in major urologic surgery: our initial experience. J Endourol.

[14] Gueli Alletti S, Chiantera V, Arcuri G (2022). Introducing the new surgical robot HUGO^TM^ RAS: system description and docking settings for gynecological surgery. Front Oncol.

[15] Monterossi G, Pedone Anchora L, Gueli Alletti S (2022). The first European gynaecological procedure with the new surgical robot HugoTM RAS. a total hysterectomy and salpingo-oophorectomy in a woman affected by BRCA-1 mutation. Facts Views Vis Obgyn.

[16] Raffaelli M, Voloudakis N, Pennestrì F (2023). Feasibility of Roux-en-Y gastric bypass with the novel robotic platform HUGO^TM^ RAS. Front Surg.

[17] Pietro BP, Salaj A, Rocco B, Formisano G (2023). First worldwide report on Hugo RAS^TM^ surgical platform in right and left colectomy. Updates Surg.

[18] Palmisano S, Giuricin M, Casagranda B, de Manzini N (2014). Zero frequency of internal hernias after laparoscopic double loop gastric bypass without closure of mesenteric defects. Surg Today.

[19] Pennestrì F, Gallucci P, Prioli F (2019). Barbed vs conventional sutures in bariatric surgery: a propensity score analysis from a high-volume center. Updates Surg.

[20] Pennestrì F, Prioli F, Sessa L (2019). Early Routine Upper Gastrointestinal Contrast Study Following Bariatric Surgery: an indispensable postoperative care or a medicolegal heritage?. Obes Surg.

[21] Dindo D, Demartines N, Clavien PA (2004). Classification of surgical complications: a new proposal with evaluation in a Cohort of 6336 patients and results of a survey. Ann Surg.

[22] Pennestrì F, Sessa L, Prioli F (2023). Robotic vs laparoscopic approach for single anastomosis duodenal-ileal bypass with sleeve gastrectomy: a propensity score matching analysis. Updates Surg.

[23] Pennestrì F, Sessa L, Prioli F (2022). Single anastomosis duodenal-ileal bypass with sleeve gastrectomy (SADI-S): experience from a high-bariatric volume center. Langenbecks Arch Surg.

[24] Himpens J, Leman G, Cadiere GB (1998). Telesurgical laparoscopic cholecystectomy. Surg Endosc.

[25] Boggi U, Vistoli F, Amorese G (2021). Twenty years of robotic surgery: a challenge for human limits. Updates Surg.

[26] Intuitive Surgical, Inc. -AnnualReports.com. https://www.annualreports.com/Company/intuitive-surgical-inc. Accessed 28 Mar 2023

[27] Fantola G, Moroni E, Runfola M (2022). Controversial role of robot in primary and revisional bariatric surgery procedures: review of the literature and personal experience. Front Surg.

[28] Soomro NA, Hashimoto DA, Porteous AJ (2020). Systematic review of learning curves in robot-assisted surgery. BJS Open.

[29] Awad S, Aguilo R, Agrawal S, Ahmed J (2015). Outcomes of linear-stapled versus hand-sewn gastrojejunal anastomosis in laparoscopic Roux en-Y gastric bypass. Surg Endosc.

[30] Jiang HP, le Lin L, Jiang X, Qiao HQ (2016). Meta-analysis of hand-sewn versus mechanical gastrojejunal anastomosis during laparoscopic Roux-en-Y gastric bypass for morbid obesity. Int J Surg.

[31] Wesley Vosburg R, Haque O, Roth E (2022). Robotic vs. laparoscopic metabolic and bariatric surgery, outcomes over 5 years in nearly 800,000 patients. Obes Surg.

[32] Murata Y, Tanemura A, Kato H (2017). Superiority of stapled side-to-side gastrojejunostomy over conventional hand-sewn end-to-side gastrojejunostomy for reducing the risk of primary delayed gastric emptying after subtotal stomach-preserving pancreaticoduodenectomy. Surg Today.

[33] Kravetz AJ, Reddy S, Murtaza G, Yenumula P (2011). A comparative study of handsewn versus stapled gastrojejunal anastomosis in laparoscopic Roux-en-Y gastric bypass. Surg Endosc.

[34] Hagen ME, Jung MK, Fakhro J (2018). Robotic versus laparoscopic stapling during robotic Roux-en-Y gastric bypass surgery: a case-matched analysis of costs and clinical outcomes. Surg Endosc.

[35] Myers SR, McGuirl J, Wang J (2013). Robot-assisted versus laparoscopic gastric bypass: comparison of short-term outcomes. Obes Surg.

[36] Rao PP (2018). Robotic surgery: new robots and finally some real competition!. World J Urol.

[37] Larkins KM, Mohan HM, Gray M (2022). Transferability of robotic console skills by early robotic surgeons: a multi-platform crossover trial of simulation training. J Robot Surg.

[38] Fleming CA, Ali O, Clements JM (2022). Surgical trainee experience and opinion of robotic surgery in surgical training and vision for the future: a snapshot study of pan-specialty surgical trainees. J Robot Surg.

